# Introduction to Special Issue “The Self-Assembly and Design of Polyfunctional Nanosystems 3.0”

**DOI:** 10.3390/ijms252010966

**Published:** 2024-10-11

**Authors:** Ruslan Kashapov, Lucia Zakharova

**Affiliations:** A.E. Arbuzov Institute of Organic and Physical Chemistry, Federal Research Center Kazan Scientific Center of Russian Academy of Sciences, 8 Arbuzov Street, Kazan 420088, Russia

Stimulus-responsive systems allowing for the controlled release of drugs [1,2,3,4,5] and diagnostic markers [6,7,8,9] are attractive solutions to numerous problems faced by clinicians in biomedicine and cancer therapy. In the work of Stoikov et al. [10], deca-substituted derivatives of pillar[5]arene containing residues of L-tryptophan and L-phenylalanine were synthesized, which were used as pH-responsive drug nanocarriers. Using the MTT assay, it was found that the synthesized pillar[5]arenes demonstrated statistically significant cytotoxic activity against the MCF-7 breast adenocarcinoma and PC-3 prostate carcinoma cell lines. Upon loading the fluorescein dye onto aggregates based on the pillar[5]arene derivatives containing L-tryptophan, the dye was released in neutral (pH 7.4) and alkaline (pH 9.2) buffer solutions. However, when the dye was loaded onto pillar[5]arene-based aggregates with L-phenylalanine residues, no release occurred regardless of the pH due to the strong intermolecular interactions present. Stoikov et al. also synthesized monosubstituted pillar[5]arenes containing both amide and carboxyl functional groups [11]. Solid lipid nanoparticles were prepared using these macrocycles. Since the pillar[5]arene containing N-alkylamide moieties was in the free form due to its shorter linker length, an equimolar amount of dodecyltrimethylammonium chloride was added in order to obtain stable nanoparticles. By varying the macrocycle/surfactant ratio in the solid lipid nanoparticles, control over the surface charge of the particles was achieved, allowing for the molecular-scale preparation of porous materials that selectively interact with different types of substrates, including biopolymers.

Inorganic nanoparticles can be modified through non-covalent interactions [12,13,14,15,16]. Supramolecular interactions play a significant role in binding drugs to iron oxide nanoparticles, which are functionalized with different types of polymers, including those that are cationic (polyethyleneimine), anionic (polystyrene sulfonate) and nonionic (dextran) [17]. The use of these polymers increased the stability of the nanoparticles in an aqueous environment by up to 4–6 times compared to that of unmodified magnetite. The particles functionalized with polyethyleneimine exhibited the highest loading capacity for doxorubicin, but the maximum release of this drug at pH 5 of up to 30% was observed from the particles functionalized with polystyrene sulfonate. Evaluating the cytotoxicity of these nanoparticles against the Neuro2A cell line showed they had no significant effect, as the survival rate of the cells remained above 80%. This indicates their high biocompatibility and potential for use in medical applications.

Due to supramolecular self-assembly, various nanomaterials possess an extremely extended surface area and uniform porosity [18,19,20,21]. The review by Eid et al. highlighted the synthesis of porous nanostructures based on graphitic carbon nitride with non-metallic dopants for photocatalytic hydrogen production [22]. The supramolecular self-assembly of multiple nitrogen-rich carbon precursors, such as melamine with cyanuric acid and urea with ammonium iodide, is an effective way to create porous nanostructures with a high surface area. This process also allows for the in situ doping of non-metals during the self-assembly process, namely, S-doping in the case of thiourea self-assembly, O-doping using cyanuric acid, and B-doping using boric acid.

Supramolecular interactions play a significant role in sensor development [23,24,25,26]. Antina et al. demonstrated the high sensitivity of the fluorescent characteristics of BODIPY dimers to the presence of polar solvents [27]. The intense fluorescence of these substances in non-polar or low-polar solvents fades sharply in polar media, such as acetone, DMF, and DMSO. The main reason for this fluorescence quenching is the specific solvation of the dyes by electron donor molecules in the solvent, leading to the formation of stable supramolecular structures. These results suggest the potential for developing fluorescent probes based on BODIPY dimers for detecting electron donor compounds in solutions and biological systems.

Gels [28,29,30] and lyotropic liquid crystals [31,32,33] are unique self-assembling systems with immense potential for both fundamental and applied research. Galyametdinov et al. optimized the formation of biocompatible gels and lyotropic liquid crystals at certain concentrations of chitosan and lactic acid [34]. The resulting liquid crystals showed the most prolonged release rates of riboflavin compared to the gels. These proposed biocompatible systems show potential as supramolecular platforms for transdermal drug delivery and controlled drug release.

The non-covalent binding of nucleic acids is essential for the development of non-viral vectors [35,36] and biosensors [23,25,37], as well as biochips [38]. Noskov et al. were the first to investigate the penetration of DNA into a lysozyme layer deposited on an aqueous subphase [39]. Despite the formation of DNA/lysozyme complexes in the surface layer, which was not observed with DNA penetration into a layer of synthetic cationic polyelectrolyte poly(N,N-diallyl-N-hexyl-N-methylammonium) chloride, a regular continuous network was not formed at the water–air interface, i.e., the DNA/lysozyme complexes were separated in the surface layer. In the case of a mixture of DNA and a synthetic polyelectrolyte, a network of aggregates formed. The supramolecular interactions between the DNA and lysozyme molecules led to the formation of folds and ridges in the mixture. This process is likely due to weaker interactions between the lysozyme and duplex DNA, as well as the stabilization of the loops of unpaired nucleotides at high local lysozyme concentrations on the surface. The interaction between DNA and the lysozyme layer is weaker compared to when DNA penetrates into synthetic polyelectrolyte layers. In high local concentrations, lysozyme predominantly interacts with non-canonical structures of DNA in the surface layer, stabilizing them and leading to the formation of more disordered aggregates.

The most popular building blocks involved in supramolecular self-assembly are surfactants [40]. In the study by Zakharova et al. [41], the antimicrobial, membranotropic and cytotoxic properties of dicationic imidazolium surfactants with alkyl groups and spacer fragments of variable lengths were investigated and compared with those of monocationic analogues. Among the dicationic surfactants, the decyl derivative showed the highest level of antimicrobial activity, whereas, for the monocationic ones, the maximum activity was observed in the case of the tetradecyl derivative. The cytotoxic effect of the monocationic imidazolium surfactants increased with the length of the alkyl chain, reaching a maximum for the hexa- and octadecyl homologues. Conversely, the imidazolium gemini surfactants did not exhibit a high level of cytotoxicity or hemolytic activity. The antimicrobial effect of these compounds was not due to membrane destruction, but rather specific interactions with cellular components. It is important to note that these surfactants exhibit highly selective effects on microbial cells, posing minimal impacts on healthy human cells. Their selectivity index varies between 30 and 100. According to these results, we suggest that these surfactants can be used for the targeted design of bioactive amphiphilic compounds for the development of new antimicrobial agents.

In summary, supramolecular self-assembly has gained the attention of researchers around the world because it allows for the spontaneous creation of porous nanostructures, sensors, gene expression vectors, antimicrobial agents and drug delivery systems. A wide range of organic and inorganic molecules can be used in this process, as shown by the research presented in this Special Issue. The spontaneous organization of these molecules enables the creation of supramolecular nanostructures under green chemistry conditions, which is a cutting-edge approach in medicine and ecology.

## References

[1] Zaborova O.V., Timoshenko V.A., Nardin C., Filippov S.K. (2023). New insights on the release and self-healing model of stimuli-sensitive liposomes. J. Colloid. Interface Sci..

[2] Grinberg V.Y., Burova T.V., Grinberg N.V., Buyanovskaya A.G., Khokhlov A.R., Kozhunova E.Y., Vyshivannaya O.V., Nasimova I.R. (2020). Functionalized thermoresponsive microgels based on N-isopropylacrylamide: Energetics and mechanism of phase transitions. Eur. Polym. J..

[3] Burova T.V., Grinberg V.Y., Grinberg N.V., Dubovik A.S., Tikhonov V.E., Orlov V.N., Plashchina I.G., Alvarez-Lorenzo C., Khokhlov A.R. (2020). Biodegradable thermoresponsive oligochitosan nanoparticles: Mechanisms of phase transition and drug binding-release. Int. J. Biol. Macromol..

[4] Poryvaev A.S., Yazikova A.A., Polyukhov D.M., Chinak O.A., Richter V.A., Krumkacheva O.A., Fedin M.V. (2021). Guest Leakage from ZIF-8 Particles under Drug Delivery Conditions: Quantitative Characterization and Guest-Induced Framework Stabilization. J. Phys. Chem C..

[5] Nikolaev A.L., Zelenko V.L., Chicherin D.S., Gopin A.V., Bozhevol’nov V.E. (2014). Thermoresponsive hydrogels with ultrasound-controlled properties. Russ. J. Gen. Chem..

[6] Li Z., Zhu D., Hui Q., Bi J., Yu B., Huang Z., Hu S., Wang Z., Caranasos T., Rossi J. (2021). Injection of ROS-Responsive Hydrogel Loaded with Basic Fibroblast Growth Factor into the Pericardial Cavity for Heart Repair Advanced Functional Materials. Adv. Funct. Mater..

[7] Zhang P., Tong Y., Huang X., Chen Y., Li Y., Luan D., Li J., Wang C., Li P., Du L. (2023). The Dual-Response-Single-Amplification Fluorescent Nanomachine for Tumor Imaging and Gastric Cancer Diagnosis. ACS Nano.

[8] Sihag P., Sagwal V., Kumar A., Balyan P., Mir R.R., Dhankher O.P., Kumar U. (2021). Discovery of miRNAs and Development of Heat-Responsive miRNA-SSR Markers for Characterization of Wheat Germplasm for Terminal Heat Tolerance Breeding. Front. Genet..

[9] Tang B., Zhang H., Cheng C., Jiang H., Bai L., Ba X., Wu Y. (2021). Development of halloysite nanotube-based hydrogel with colorimetric H_2_O_2_-responsive character. Appl. Clay Sci..

[10] Sultanaev V., Yakimova L., Nazarova A., Mostovaya O., Sedov I., Davletshin D., Gilyazova E., Bulatov E., Li Z.-T., Zhang D.-W. (2023). Decasubstituted Pillar[5]arene Derivatives Containing L-Tryptophan and L-Phenylalanine Residues: Non-Covalent Binding and Release of Fluorescein from Nanoparticles. Int. J. Mol. Sci..

[11] Nazarova A., Yakimova L., Filimonova D., Stoikov I. (2022). Surfactant Effect on the Physicochemical Characteristics of Solid Lipid Nanoparticles Based on Pillar[5]arenes. Int. J. Mol. Sci..

[12] Sheng Y., Suhartono T., Hazmatulhaq F., Kamil M.P., Assfour B., Al Zoubi W., Ko Y.G. (2024). Conjugation of defect-controlled nucleation and self-assembly growth achieving uniform amelioration of organic micro-flowers: Surface properties and DFT analysis. J. Chem. Eng..

[13] Priya L., Mehta S., Gevariya D., Sharma R., Panjwani D., Patel S., Ahlawat P., Dharamsi A., Patel A. (2024). Quantum Dot-based Bio-conjugates as an Emerging Bioimaging Tool for Cancer Theranostic—A Review. Curr. Drug Targets.

[14] Bansal K., Devi N., Aqdas M., Kumar M., Agrewala J.N., Katare O.P., Sharma R.K., Wangoo N. (2023). Inorganic gold nanoparticles-TAT hybrid for the effective delivery of doxorubicin into cancer cells. J. Drug Deliv. Technol..

[15] Zhou W.-L., Chen Y., Lin W., Liu Y. (2021). Luminescent lanthanide-macrocycle supramolecular assembly. Chem. Commun..

[16] Uniyal S., Choudhary K., Sachdev S., Kumar S. (2024). Nano-bio fusion: Advancing biomedical applications and biosensing with functional nanomaterials. Opt. Laser Technol..

[17] Khabibullin V.R., Chetyrkina M.R., Obydennyy S.I., Maksimov S.V., Stepanov G.V., Shtykov S.N. (2023). Study on Doxorubicin Loading on Differently Functionalized Iron Oxide Nanoparticles: Implications for Controlled Drug-Delivery Application. Int. J. Mol. Sci..

[18] Nugmanova A.G., Kalinina M.A. (2022). Supramolecular Self-Assembly of Hybrid Colloidal Systems. Colloid J..

[19] Shaukat A., Anaya-Plaza E., Beyeh N.K., Kostiainen M.A. (2022). Simultaneous Organic and Inorganic Host-Guest Chemistry within Pillararene-Protein Cage Frameworks. Chem. Eur. J..

[20] Song Y., Zhou C., Zheng Z., Sun P., She Y., Huang F., Mo Z., Yuan J., Li H., Xu H. (2023). Porous carbon nitride nanotubes efficiently promote two-electron O_2_ reduction for photocatalytic H_2_O_2_ production. J. Alloys Compd..

[21] Bertram C., Miller D.P., Schunke C., Kemeny I., Kimura M.W., Bovensiepen U., Zurek E., Morgenstern K. (2022). Interplay of Halogen and Weak Hydrogen Bonds in the Formation of Magic Nanoclusters on Surfaces. J. Phys. Chem C..

[22] Lu Q., Abdelgawad A., Li J., Eid K. (2022). Non-Metal-Doped Porous Carbon Nitride Nanostructures for Photocatalytic Green Hydrogen Production. Int. J. Mol. Sci..

[23] Shamagsumova R.V., Shurpik D.N., Kuzin Y.I., Stoikov I.I., Rogov A.M., Evtugyn G.A. (2022). Pillar[6]arene: Electrochemistry and application in electrochemical (bio)sensors. J. Electroanal. Chem..

[24] Traven V.F., Cheptsov D.A., Lodeiro C. (2023). Control of Fluorescence of Organic Dyes in the Solid-State by Supramolecular Interactions. J. Fluoresc..

[25] Parfenov A.A., Mumyatov A.V., Sagdullina D.K., Shestakov A.F., Troshin P.A. (2021). Ammonia gas sensors using 1,4,5,8,9,11-hexaazatriphenylene hexacarbonitrile semiconductor films. Synth. Met..

[26] Artemenko A.A., Burilov V.A., Solov’eva S.E., Antipin I.S. (2022). Covalent and Supramolecular Conjugates of Calixarenes with Some Fluorescent Dyes of the Xanthene Series. Colloid J..

[27] Kalyagin A., Antina L., Ksenofontov A., Antina E., Berezin M. (2022). Solvent-Dependent Fluorescence Properties of CH2-bis(BODIPY)s. Int. J. Mol. Sci..

[28] Zenikov G.R., Khizhnyak S.D., Ivanova A.I., Pakhomov P.M. (2024). The Self-Organization and Gelation Processes in a Cysteine–Silver Solution Containing Chitosan and an Electrolyte. Colloid J..

[29] Akhmetshin S.R., Larionov R.A., Klimovitskii A.E., Skvortsova P.V., Akhmadiyarov A.A., Ziganshina S.A., Gorbatchuk V.A., Ziganshin M.A. (2023). Peculiarities of some Fmoc-dipeptides gelation in DMSO/water medium. J. Mol. Liq..

[30] Zuev Y.F., Derkach S.R., Bogdanova L.R., Voron’ko N.G., Kuchina Y.A., Gubaidullin A.T., Lunev I.V., Gnezdilov O.I., Sedov I.A., Larionov R.A. (2023). Underused Marine Resources: Sudden Properties of Cod Skin Gelatin Gel. Gels.

[31] Ilincă T.A., Iliș M., Micutz M., Cîrcu V. (2023). Liquid Crystalline and Gel Properties of Luminescent Cyclometalated Palladium Complexes with Benzoylthiourea Ligands. Gels.

[32] Guo W., Wang R., Liu L., Xie L., Feng Y., Yin H. (2024). Fabricating supramolecular lyotropic crystals at subzero temperatures: The pivotal role of antifreeze and nanostructures. J. Mol. Liq..

[33] Yin H., Guo W., Wang R., Doutch J., Li P., Tian Q., Zheng Z., Xie L., Feng Y. (2024). Self-Assembling Anti-Freezing Lamellar Nanostructures in Subzero Temperatures. Adv. Sci..

[34] Selivanova N.M., Galeeva A.I., Galyametdinov Y.G. (2022). Chitosan/Lactic Acid Systems: Liquid Crystalline Behavior, Rheological Properties, and Riboflavin Release In Vitro. Int. J. Mol. Sci..

[35] Finotti A., Gasparello J., Casnati A., Corradini R., Gambari R., Sansone F. (2021). Delivery of Peptide Nucleic Acids Using an Argininocalix[4]arene as Vector. Methods Mol. Biol..

[36] Miele D., Xia X., Catenacci L., Sorrenti M., Rossi S., Sandri G., Ferrari F., Rossi J.J., Bonferoni M.C. (2021). Chitosan Oleate Coated PLGA Nanoparticles as siRNA Drug Delivery System. Pharmaceutics.

[37] Wang Y., Di S., Yu J., Wang L., Li Z. (2023). Recent advances of graphene-biomacromolecule nanocomposites in medical applications. J. Mater. Chem. B.

[38] Juru A.U., Cai Z., Jan A., Hargrove A.E. (2020). Template-guided selection of RNA ligands using imine-based dynamic combinatorial chemistry. Chem. Commun..

[39] Chirkov N.S., Lin S.-Y., Michailov A.V., Miller R., Noskov B.A. (2022). DNA Penetration into a Lysozyme Layer at the Surface of Aqueous Solutions. Int. J. Mol. Sci..

[40] Movchan T.G., Rusanov A.I., Plotnikova E.V. (2022). Solubilization of nile red in aqueous solutions of tetradecyltrimethylammonium bromide. Russ. J. Gen. Chem..

[41] Amerkhanova S.K., Voloshina A.D., Mirgorodskaya A.B., Lyubina A.P., Kuznetsova D.A., Kushnazarova R.A., Mikhailov V.A., Zakharova L.Y. (2021). Antimicrobial Properties and Cytotoxic Effect of Imidazolium Geminis with Tunable Hydrophobicity. Int. J. Mol. Sci..

